# Crowdsourced dataset to study the generation and impact of text highlighting in classification tasks

**DOI:** 10.1186/s13104-019-4858-z

**Published:** 2019-12-21

**Authors:** Jorge Ramírez, Marcos Baez, Fabio Casati, Boualem Benatallah

**Affiliations:** 1 Department of Information Engineering and Computer Science, University of Trento, Via Sommarive, 9, Povo, 38123 Trento, TN Italy; 2 School of Engineering Entrepreneurship, Tomsk Polytechnic University, Tomsk, Russia; 3 School of Computer Science and Engineering, University of New South Wales, Sydney, Australia

**Keywords:** Crowdsourcing, Text highlighting, Classification, Question answering, Extractive summarization

## Abstract

**Objectives:**

Text classification is a recurrent goal in machine learning projects and a typical task in crowdsourcing platforms. Hybrid approaches, leveraging crowdsourcing and machine learning, work better than either in isolation and help to reduce crowdsourcing costs. One way to mix crowd and machine efforts is to have algorithms highlight passages from texts and feed these to the crowd for classification. In this paper, we present a dataset to study text highlighting generation and its impact on document classification.

**Data description:**

The dataset was created through two series of experiments where we first asked workers to (i) classify documents according to a relevance question and to highlight parts of the text that supported their decision, and on a second phase, (ii) to assess document relevance but supported by text highlighting of varying quality (six human-generated and six machine-generated highlighting conditions). The dataset features documents from two application domains: systematic literature reviews and product reviews, three document sizes, and three relevance questions of different levels of difficulty. We expect this dataset of 27,711 individual judgments from 1851 workers to benefit not only this specific problem domain, but the larger class of classification problems where crowdsourced datasets with individual judgments are scarce.

## Objective

In this paper, we introduce datasets derived from multiple crowdsourcing experiments for document classification tasks. These experiments resemble a two-step pipeline that first highlights relevant passages and then classifies the documents. The datasets include the individual judgments provided by the workers for both steps of our pipeline, totaling 27,711 judgments from 1851 workers.

Research has shown the feasibility of leveraging non-expert annotators in complex NLP tasks [1]. Text classification, in particular, is a recurrent goal of machine learning (ML) projects, and a typical task in crowdsourcing platforms. Hybrid approaches, combining ML and crowd efforts, have been proposed to boost accuracy and reduce costs [2–4]. One possibility is to use automatic techniques for highlighting relevant excerpts in the text and then ask workers to classify. And in doing so, workers could rely on the highlights, and avoid reading parts of the text, or ignore the highlighting and read the full text. In this context, we run crowdsourcing experiments to study the effects that text highlighting has on human performance in classification tasks [5]. In these experiments, we focused on two crowdsourcing tasks: gathering the text highlights, and classification. The highlighting gathering task produced a dataset containing crowd-generated highlights that could serve, for example, researchers in studying automatic techniques such as text summarizers and question-answering models. The classification datasets could benefit researchers from the human computation community working on problems such as assessing and assuring quality [6], budget optimization [7, 8], and worker behavior [9], as well as further investigating highlighting support.

## Data description

In the following we described the crowdsourcing experiments that generated the dataset as well as the dataset structure.

### Task

In our experiments, we asked workers to assess whether a document is relevant to a given question (predicate), augmenting the task design found in the literature [10, 11]. The documents come from two different domains systematic literature reviews (SLR) and amazon reviews. For the SLR domain, we considered two predicates *“Does the paper describe a study that involves older adults (60+)?”* (OA), and *“Does the paper describe a study that involves technology for online social interactions?”* (Tech). For Amazon reviews, we asked, *“Is this review written on a book?”* (AMZ).

All tasks were run in the crowdsourcing platform Figure Eight (https://www.figure-eight.com/). And personal information was not requested to workers; we only collected class labels and statistics related to effort.

### Gathering text highlights

The first step is to generate highlights. This step serves as the basis of our study on text highlighting as an aid to workers in the classification tasks. We considered crowdsourcing and ML to generate the highlighted excerpts. For crowd-generated highlights, we asked workers to classify documents and to justify their decisions by highlighting passages from the text. For machine-generated highlights we used state-of-the-art extractive summarization and question-answering models. Two experts judged the quality of the highlights provided by the crowd and automatic techniques (Kappa was 0.87 for OA, 0.72 for Tech and 0.66 for AMZ). Table 1 shows the files containing the generated highlights (crowd and ML); both datasets include the individual highlights and associated quality.

**Table 1:** Overview of data files/data sets

Label	Name of data file/data set	File types (file extension)	Data repository and identifier (DOI or accession number)
Crowd highlights	crowdsourced_highlights.csv: the dataset containing highlighted passages provided by workers from Figure Eight	Comma-separated values (.csv)	10.6084/m9.figshare.9917162.v4
ML highlights	ml_highlights.csv: the dataset containing the highlighted passages produced by automatic techniques	Comma-separated values (.csv)	10.6084/m9.figshare.9917162.v4
Classification OA crowd highlights	classification_oa-crowd-highlights.csv: first dataset from Experiment 1. OA predicate using crowd-generated highlights	Comma-separated values (.csv)	10.6084/m9.figshare.9917162.v4
Classification tech crowd highlights	classification_tech-crowd-highlights.csv: second dataset from Experiment 1. Tech predicate using crowd-generated highlights	Comma-separated values (.csv)	10.6084/m9.figshare.9917162.v4
Classification Amazon crowd highlights	classification_amazon-crowd-highlights.csv: third dataset from Experiment 1. AMZ predicate using crowd-generated highlights	Comma-separated values (.csv)	10.6084/m9.figshare.9917162.v4
Classification tech 3 × 12 crowd highlights	classification_tech-3 × 12-crowd-highlights.csv: first dataset from Experiment 2. tech predicate using crowd-generated highlights. Layout 3 × 12	Comma-separated values (.csv)	10.6084/m9.figshare.9917162.v4
Classification tech 6 × 6 crowd highlights	classification_tech-6 × 6-crowd-highlights.csv: second dataset from Experiment 2. tech predicate using crowd-generated highlights. layout 6 × 6	Comma-separated values (.csv)	10.6084/m9.figshare.9917162.v4
Classification OA ML highlights	classification_oa-ML-highlights.csv: first dataset from Experiment 3. OA predicate using machine-generated highlights	Comma-separated values (.csv)	10.6084/m9.figshare.9917162.v4
Classification Tech ML highlights	classification_tech-ML-highlights.csv: second dataset from Experiment 3. Tech predicate using machine-generated highlights	Comma-separated values (.csv)	10.6084/m9.figshare.9917162.v4
Classification Amazon ML highlights	classification_amazon-ML-highlights.csv: third dataset from Experiment 3. AMZ predicate using machine-generated highlights	Comma-separated values (.csv)	10.6084/m9.figshare.9917162.v4

### Classification with highlighting support

#### Experiment 1

In this experiment, we asked workers to classify documents, giving additional support by highlighting passages from the text. Workers proceeded on pages of three documents each, up to six pages (3 × 6 layout). We categorized the available crowdsourced highlights according to their quality and derived six experimental conditions for our study. The baseline condition does not show any highlighted text. The 0%, 33%, 66% and 100% show highlights of varying quality. For example, on a page with three documents, the 33% condition shows one high-quality highlight and two low-quality ones. Finally, the aggregation condition combines multiple highlights similar to aggregating votes in crowdsourcing tasks.

#### Experiment 2

This experiment focused on longer documents and pages, using 6 × 6 and 3 × 12 layouts and crowd-generated highlights. We keep the baseline as one experimental condition, and we introduce the 83% quality as the other.

#### Experiment 3

This experiment used machine-generated highlights, using a 3 × 6 layout and six experimental conditions: BertSum, Refresh, Bert-QA, AggrML, 100%ML, baseline. BertSum [12] and Refresh [13], are extractive summarization techniques, while Bert-QA [14] is a question-answering model. AggrML aggregates the output from the three algorithms, and 100%ML only uses machine-generated highlighting assessed by experts as being of good quality.

We encourage readers to check [5] for a more in-depth explanation of the experimental settings. Table 1 overviews the available datasets derived from our experiments.

## Limitations

The dataset described in this paper features a set of dimensions that allow for an exploration of approaches, but that cannot be considered comprehensive. The dataset is still limited to two types of classification tasks, includes only the most widely used state-of-the-art algorithms for highlight generation, and relies on two task designs for crowd classification. Besides, the experiments with longer pages and documents (Experiment 2) are extensions of the first experiment and focus only on one relevance question.

These alternatives have been carefully selected, but more systematic studies will require a more in-depth investigation of each of these dimensions.

## Data Availability

The data described in this Data note can be freely and openly accessed on Figshare [15]. Please see Table 1 and reference list for details and links to the data.

## Abbreviations

ML: machine learning
SLR: systematic literature reviews
OA: relevance question: “Does the paper describe a study that involves older adults (60+)?*”*
Tech: relevance question: “Does the paper describe a study that involves technology for online social interactions?”
AMZ: relevance question: “Is this review written on a book?”

## References

[1] Snow R, O’Connor B, Jurafsky D, Ng AY. Cheap and fast—but is it good? Evaluating non-expert annotations for natural language tasks. In: 2008 conference on empirical methods in natural language processing, EMNLP 2008, proceedings of the conference, 25–27 October 2008, Honolulu, Hawaii, USA, a meeting of SIGDAT, a special interest group of the ACL. 2008. p. 254–63. http://www.aclweb.org/anthology/D08-1027.

[2] Cheng J, Bernstein MS. Flock: hybrid crowd-machine learning classifiers. In: CSCW 2015. 2015. 10.1145/2675133.2675214.

[3] Wallace BC, Noel-Storr A, Marshall IJ, Cohen AM, Smalheiser NR, Thomas J (2017). Identifying reports of randomized controlled trials (RCTs) via a hybrid machine learning and crowdsourcing approach. JAMIA.

[4] Krivosheev Evgeny, Casati Fabio, Baez Marcos, Benatallah Boualem (2018). Combining Crowd and Machines for Multi-predicate Item Screening. Proceedings of the ACM on Human-Computer Interaction.

[5] Ramírez J, Baez M, Casati F, Benatallah B. Understanding the impact of text highlighting in crowdsourcing tasks. In: Proceedings of the seventh AAAI conference on human computation and crowdsourcing, HCOMP 2019, vol. 7. AAAI. 2019. p. 144–52.

[6] Daniel F, Kucherbaev P, Cappiello C, Benatallah B, Allahbakhsh M (2018). Quality control in crowdsourcing: a survey of quality attributes, assessment techniques, and assurance actions. ACM Comput Surv.

[7] Dai P, Lin CH, Weld DS (2013). POMDP-based control of workflows for crowdsourcing. Artif Intell.

[8] Lan D, Reed K, Shin A, Trushkowsky B. Dynamic filter: adaptive query processing with the crowd. In: Proceedings of the fifth AAAI conference on human computation and crowdsourcing, HCOMP 2017, 23–26 October 2017, Quebec City, Quebec, Canada. 2017. p. 118–27. https://aaai.org/ocs/index.php/HCOMP/HCOMP17/paper/view/15932.

[9] Alagarai Sampath H, Rajeshuni R, Indurkhya B.. Cognitively inspired task design to improve user performance on crowd-sourcing platforms. In: CHI conference on human factors in computing systems, CHI’14, Toronto, ON, Canada—April 26–May 01, 2014. 2014. p. 3665–374. 10.1145/2556288.2557155.

[10] Krivosheev E, Casati F, Caforio V, Benatallah B. Crowdsourcing paper screening in systematic literature reviews. In: Proceedings of the fifth AAAI conference on human computation and crowdsourcing, HCOMP 2017, 23–26 October 2017, Quebec City, Quebec, Canada. 2017. p. 108–17

[11] Ramirez J, Krivosheev E, Baez M, Casati F, Benatallah B. Crowdrev: a platform for crowd-based screening of literature reviews. In: Collective intelligence, CI 2018. 2018.

[12] Liu Y. Fine-tune BERT for extractive summarization. arXiv preprint arXiv:1903.10318. 2019.

[13] Narayan S, Cohen SB, Lapata M. Ranking sentences for extractive summarization with reinforcement learning. In: NAACL 2018.

[14] Devlin J, Chang MW, Lee K, Toutanova K. Bert. Pre-training of deep bidirectional transformers for language understanding. arXiv preprint arXiv:1810.04805. 2018.

[15] Ramírez J, Baez M, Casati F, Benatallah B (2019). Figshare.

